# An Individual-Based Model of Transmission of Resistant Bacteria in a Veterinary Teaching Hospital

**DOI:** 10.1371/journal.pone.0098589

**Published:** 2014-06-03

**Authors:** Neeraj Suthar, Sandip Roy, Douglas R. Call, Thomas E. Besser, Margaret A. Davis

**Affiliations:** 1 Paul G. Allen School for Global Animal Health, College of Veterinary Medicine, Washington State University, Pullman, Washington, United States of America; 2 School of Electrical Engineering and Computer Science, College of Veterinary Medicine, Washington State University, Pullman, Washington, United States of America; 3 Dept. of Veterinary Microbiology and Pathology, College of Veterinary Medicine, Washington State University, Pullman, Washington, United States of America; 4 Dept. of Veterinary Clinical Sciences, College of Veterinary Medicine, Washington State University, Pullman, Washington, United States of America; University Medical Center Groningen, Netherlands

## Abstract

Veterinary nosocomial infections caused by antibiotic resistant bacteria cause increased morbidity, higher cost and length of treatment and increased zoonotic risk because of the difficulty in treating them. In this study, an individual-based model was developed to investigate the effects of movements of canine patients among ten areas (transmission points) within a veterinary teaching hospital, and the effects of these movements on transmission of antibiotic susceptible and resistant pathogens. The model simulates contamination of transmission points, healthcare workers, and patients as well as the effects of decontamination of transmission points, disinfection of healthcare workers, and antibiotic treatments of canine patients. The model was parameterized using data obtained from hospital records, information obtained by interviews with hospital staff, and the published literature. The model suggested that transmission resulting from contact with healthcare workers was common, and that certain transmission points (housing wards, diagnostics room, and the intensive care unit) presented higher risk for transmission than others (lobby and surgery). Sensitivity analyses using a range of parameter values demonstrated that the risk of acquisition of colonization by resistant pathogens decreased with shorter patient hospital stays (*P*<0.0001), more frequent decontamination of transmission points and disinfection of healthcare workers (*P*<0.0001) and better compliance of healthcare workers with hygiene practices (*P*<0.0001). More frequent decontamination of heavily trafficked transmission points was especially effective at reducing transmission of the model pathogen.

## Introduction

Antimicrobial resistance is a growing concern in modern health care settings as it increases morbidity, cost of treatment and mortality [1]. The prevalence of resistant bacteria in food animals may present a direct risk to public health [2], [3] and companion animals may act as reservoirs of antimicrobial resistant bacteria that can be transmitted directly to people [4], [5], [6], [7]. In human hospitals nosocomial infections cause approximately 90,000 deaths per year and an average of 5–10% of patients acquire nosocomial infections [8]. In veterinary hospitals the risk factors for nosocomial infections are similar to those in human healthcare settings. Lack of hand hygiene, use of invasive procedures, prolonged treatment and hospitalization and reliance on antimicrobials increase the risk of amplifying and transmitting antimicrobial resistant pathogens in veterinary hospitals [9], [10]. *Escherichia coli* and *Klebsiella* spp. in particular have been strongly associated with urinary tract infections among human patients [11], [12]. Canine cases of urinary tract infections caused by *E. coli* and *Klebsiella pneumoniae* are commonly diagnosed in veterinary settings and increasing numbers of antibiotic resistance cases in these bacterial species have made effective treatment more difficult [13].

Risk-based case control studies have shown that hospitalization is a serious risk factor for dogs becoming rectal carriers of multi-drug resistant (MDR) *E. coli* [14], [15]. Dogs staying for over 6 days experience an increased risk of carrying MDR *E. coli* while those patients who had been hospitalized previously and/or had been treated with fluoroquinolones previously had higher probability of carrying MDR *E. coli* on arrival to the hospital. Veterinary hospitals may be the major source of resistant and MDR *E. coli* in horses [16]. Furthermore, increasing prevalence of MDR bacterial colonization of companion animals may have serious public health impacts [17].

Mathematical epidemic models have been applied to human hospital settings to analyze the risk factors associated with transmission of antibiotic resistant pathogens, to study associated molecular mechanisms and to evaluate control measures [18]. Three types of models have been commonly used to track nosocomial infections: deterministic models [19], [20], [21], [22], stochastic models [23], [24], [25] and individual based models [26]. These models indicate that longer duration of treatment [26], delayed treatment and early breaks in treatment [27], reduced hospital staff [28], longer healthcare worker visits and larger populations of patients in the hospitals [29] increase the dissemination of antibiotic resistant bacteria while better hand hygiene compliance [19] and combinatorial antibiotic therapy reduces this risk [27]. Horizontal gene transfer in the context of excessive antibiotic use can also lead to increased acquisition of antibiotic resistance, thereby potentially increasing the duration of antibiotic treatment and potential for treatment failure [30], [31].

A model for veterinary settings has to account for the more frequent movement of the patients that is characteristic of these settings as compared to human hospitals. This movement is due, in part, to patient needs (e.g., environmental enrichment, walks for urination and defecation). Also, in veterinary settings there is reduced control over animal contacts with healthcare workers and surfaces due to petting, hand carriage of smaller animals, more proximity to the floor and defecation in cages. There are also important differences in housing and intensive care unit (ICU) arrangements. More canine patients can be accommodated in a much smaller veterinary hospital ICU by stacking their cages on top of each other as compared to the more spacious accommodation usually provided to human patients.

Published mathematical models for veterinary settings have been limited to deterministic approaches or regression analyses [32], [33]. While these models and similar human models are useful for predicting risk factors and evaluating intervention measures, they do not take animal movements within the hospital into account. We developed an individual-based model (IBM) that tracks the movement of patients across the different points in the veterinary hospital where they come into contact with healthcare workers and various surfaces. This model improves on previous attempts to model nosocomial spread of antibiotic resistant pathogens by including variations in the rates of surface and healthcare worker contamination, routes of patient movement in the hospital, and other biologically relevant variables. We then use it to predict the probability of spread of antibiotic resistance under different control policies and changed hospital operational conditions to identify approaches to reduce the incidence of pathogen transmission in general, and multidrug resistant pathogen transfer in particular.

## Materials and Methods

### General model

We developed a stochastic IBM that tracks colonization of individual patients with resistant and non-resistant strains of a single bacterial pathogen as the individuals move through a veterinary hospital. For this model, we assumed that the pathogen could be carried asymptomatically in the gastrointestinal tract (colonization) and in some patients cause systemic infections such as wound, bloodstream or urinary tract infections, similar to known veterinary and human nosocomial pathogens such as *E. coli*, *K. pneumoniae*, or *Enterococcus* spp. [14], [34], [35], [36]. Canine patients transit through this network model of the veterinary hospital, with a maximum of *P* patients in the hospital at any time. During their visits, each patient visits a sequence of transmission points (among *T* in total), which represent locations within the hospital where colonization can occur (e.g., surgery beds, diagnostic rooms, housing, etc). The patients are attended to by *H* human healthcare workers, each of whom is assigned to a single patient at any time. In the model, patients may be colonized by the pathogen by either contact with a contaminated transmission point or a contaminated health care worker. The model also incorporates the bacterial loads of colonized and infected patients, as well as the effects of antibiotic treatment of the infections. Specific components of the model discussed below include: 1) the temporal resolution and scope of the model, 2) intake of patients, 3) movement and care of patients in the hospital, 4) colonization and contamination, and 5) treatment.

#### Temporal Resolution and Scope

The model tracks colonization of patients by the pathogen over a long time horizon (months to years), with model dynamics resolved across several time scales. In particular, the intake of patients into the hospital and treatment with antibiotics is captured at a daily scale. Further, the day is subdivided into several multi-hour shifts (e.g., 3 shifts of eight hours each), after which health-care workers are replaced and treatment efforts re-initiated. Finally, colonization of patients and contamination of healthcare workers and transmission points is modeled at a fine resolution (time step, typically 1–15 minutes). A smaller time step size allows use of an exponential distribution to select values for various duration parameters in the model. We will refer to these different time resolutions in describing different aspects of the model.

#### Intake of Patients

New patients are taken into the hospital on a daily basis. The number of new patients is modeled as a Poisson random variable with a mean *PD*, with patients taken in up to the capacity of the hospital. Each patient taken into the hospital is in one of *Q* classes (labeled 1, … , *Q*), which reflect their treatment needs (e.g. surgery, routine visit for a checkup, special diagnostics). Each of these classes would either require hospitalization or not. Specifically, each patient is modeled as being in class q ε 1, … , *Q* with probability *p_q_*, independently of the other patients. The duration that each hospitalized patient in a class *q* remains in the hospital (or the time of visit of the patient) is modeled as an independent exponential random variable, with mean *v_q_*. This duration is specified according to the stochastic model at the time of intake. Each incoming patient may be pre-colonized with resistant, non-resistant, or both strains at small probabilities, independently of the other patients. As soon as the patient is admitted to the hospital a healthcare worker is assigned to that patient.

#### Patient Movement and Care

Each patient is modeled as following a route through the hospital, i.e. transitioning through a sequence of transmission points during its time at the hospital. Specifically, the routes followed by the patients in each of the *Q* classes are governed by distinct stochastic-sequence models: for instance, a regular-checkup patient may only transition among the hospital lobby, exam room, and diagnostics facility, while a surgery patient visits a larger number of transmission points (e.g., housing, operating rooms, etc). The route sequence can have both complex pre-determined transitions (to account for restricted movement during certain times of day) or simpler randomized transitions. As each patient follows its route, the patient remains at each transmission point for a stochastically-determined time-duration. Specifically, the patient remains in the transmission point for an exponentially-distributed time with average stay duration of *TAV_t_* depending on the transmission point *t*, or until the patient's hospital-visit duration is exceeded. Once the healthcare worker's assignment to the patient is completed, he/she is immediately re-assigned to any unassigned patient with equal probability (or is re-assigned as soon as a patient becomes available); the health-care worker continues to transition among patients in this fashion. The healthcare worker remains with each patient for an exponentially distributed duration with mean duration given by *AV*, unless the assigned patient leaves the hospital.

#### Colonization, Infection and Contamination

Each patient is in one of four colonization states at each time-step during its visit to the hospital: uncolonized (U), colonized with a non-resistant strain of pathogen (NR), colonized with a resistant strain of pathogen (R) or colonized with both resistant and non-resistant strains of pathogen (NR+R). For each colonized patient, the model also captures the patient's bacterial loads for the non-resistant and resistant strains, and determines infection status. A subset of colonized patients become infected with the colonizing strain; in the model each colonized patient develops an infection with probability *PI*. Similarly, each transmission point and each health care worker may be classified into four contamination categories: uncontaminated, contaminated with the non-resistant strain, contaminated with the resistant strain, or contaminated with both strains. Broadly, patients may become colonized due to contact with either contaminated healthcare workers or contaminated transmission points. Further, colonized patients may contaminate both healthcare workers and transmission points, and there may also be direct cross-contamination between health-care-workers and transmission points.

Within this system of movement, a patient can be colonized with an initial arbitrary bacterial load due to a contaminated healthcare worker and/or transmission point per visit with a probability of *PC* with two provisos. First, the probability of contamination of the transmission point and/or the healthcare worker per patient visit, with the strain from a colonized patient, is directly proportional to the patients' bacterial load and inversely proportional to the surface area of the transmission point. Healthcare worker and transmission point can also cross-contaminate with a probability of *PC.* Contaminated healthcare workers are disinfected and contaminated transmission points are decontaminated with a probability *DE* after time intervals that are exponentially distributed with an average decontamination time *AC*. Secondly, the bacterial load of the colonized patient increases in absence of antibiotic therapy and is updated at the end of every shift. The simultaneous evolution of resistant and non-resistant bacterial loads is derived from a model previously described as equation 2 by Webb *et al.* [37] which simulates transfer of resistance plasmids from plasmid-bearing to non-plasmid bearing bacteria.

#### Treatment

The probability that a colonized patient becomes infected (i.e., symptomatic of disease) is *PI* and the probability that an infection is detected at the end of shift is *DR* (detection rate). Patients detected with an infection are given a primary antibiotic treatment immediately, which initiates a decrease in the load of non-resistant strains every shift. After antibiotic susceptibility information is available, the treatment is suitably modified to reduce any resistant strain load carried by the patient as well. When the bacterial load of colonized patients goes below an arbitrary recovery threshold, they become “uncolonized.”

### Model for the Washington State University Veterinary Teaching Hospital (WSU-VTH)

We modelled the transmission of antibiotic resistant enteric bacteria among canine patients at the WSU-VTH. Data for parameterization of the model was drawn in part from hospital infection control surveillance activities. This surveillance involved collection of individual rectal swab samples from canine patients in three small animal services (intensive care, surgery and neurology) between September, 2009 and April, 2013. Data including antibiotic treatments, which services the animal visited, and the number of days in the hospital were recorded at the time of sample collection. Fecal swabs were plated directly onto MacConkey agar supplemented with ampicillin (16 ug/ml) and nalidixic acid (32 ug/ml) to select for Gram-negative bacteria that were resistant to both of these antibiotics. Any growth was noted and isolated colonies were submitted to the Washington State Animal Disease Diagnostic Laboratory (WADDL) for bacterial species identification. The average number of different categories of patients visiting each day and their duration of stay was calculated using this surveillance data and computerized hospital medical records. Each new patient was classified into one of the three categories: (i) surgery including elective, non-elective and emergency surgery; (ii) non-surgical disease including infectious disease, inflammatory disease, metabolic and other chronic diseases; or (iii) regular check-up involving routine visits for physical exams and vaccinations.

**Figure 1:**
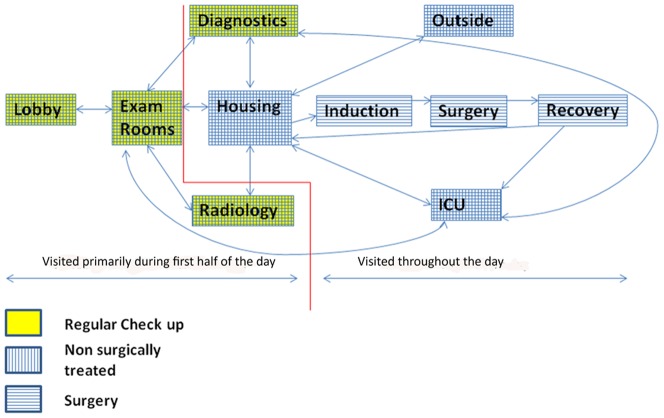
Patient movement inside the hospital. Patients seen for regular exams (yellow) are limited to the lobby, diagnostics and radiology. Patients seen for non-surgical problems (vertical lines) may be housed in wards or in the ICU and are taken outside for walks. Patients coming to WSU VTH for surgery (horizontal lines) have additional movements to the induction, surgery and recovery rooms.

Based on the category assigned in the model, the patients will have different average lengths of stay (surgery and non-surgical disease, 5 days; regular check-up, 0.5 day) and follow different routes in the hospital (Fig. 1). We considered ten areas in the hospital that canine patients may visit during their hospital stay as potential transmission points. These included the lobby, the exam rooms, the diagnostics room (diagnosis of patients is done here and in rare cases patients stay overnight), radiology, the housing wards (a large area with kennels for hospitalization), the outside dog-walking area, the ICU, the induction room (patients are prepared for surgery here), the surgery rooms, and the recovery room (patients have a transition stay here after surgery before being moved back to housing or ICU). At this hospital most elective surgeries are performed during the morning hours and for the most part patient movement during the night is limited to housing, ICU and diagnostics areas, therefore those movement constraints are included in the model.

We assumed that 10% of the daily new arrivals are colonized with a non-resistant strain and an additional 3% are colonized with the resistant strain at the time of entry into the hospital. The number of patients visiting daily averages 24.32. Baseline parameters for average time spent at each transmission point, average number of healthcare workers present in the hospital at any given time, and the maximum hospitalized patient load were based on information from hospital staff. The time required by WADDL to report antibiotic resistance profiles for hospitalized patients was most often 2 days (range, 1–8 days), and this was used as the time before treatment modification in the model. Baseline parameter values are given in Table 1.

**Table 1:** List of parameters and their baseline values.

Parameter name	Base level value in our model	Description
PD	24.372 per day***	Mean number of patients visiting the hospital daily
H	25*	Number of healthcare workers at any time
P	100*	Maximum number of patients in the hospital
T	10	Transmission points considered
Q	3	Routes considered
TAV_1_	30 min*	Average time spent in the lobby at a time
TAV_2_	120 min*	Average time spent in the exam room at a time
TAV_3_	300 min*	Average time spent in the Diagnostics at a time
TAV_4_	30 min*	Average visit time at Radiology
TAV_5_	600 min*	Average visit time at Housing
TAV_6_	600 min*	Average visit time at ICU
TAV_7_	30 min*	Average visit time outside
TAV_8_	120 min*	Average visit time at Induction room
TAV_9_	60 min*	Average visit time at Surgery
TAV_10_	120 min*	Average visit time at Recovery
AV	60 min**	Average time of healthcare worker visit
AC	60 min**	Average time before disinfection/decontamination of HCW/TP
PC	0.06**	Probability of colonization of patient given contact
V_1,2_	5 days***	Average length of stay of surgery and non-surgical treatment patients
V_3_	½ day*	Average length of stay of regular check-up patients
DE	0.9*	Probability of disinfection/decontamination of HCW/TP at the end of contamination period
PI	0.3*	Probability that a colonized patient becomes infected
DR	0.8*	Probability that an infection is detected at the end of shift
p_1_	0.211***	Fraction of patients seen at WSU VTH that go to surgery
p_2_	0.022***	Fraction of patients coming to WSU VTH for non-surgical or disease treatment
p_3_	0.767***	Fraction of patients coming for routine exams at WSU VTH

*values for the WSU VTH were based on information from the hospital staff.

**values used by D'Agata et. al, 2007 [26], in the IBM for human patients.

***values estimated in this study using the hospital records and surveillance data.

HCW- healthcare worker, TP- transmission point.

### Simulations

Model code was developed using MATLAB vR2012a (Mathworks, Natick, Massachusetts). Simulations begin with an empty hospital and continue over a period of one year. Five hundred simulations using baseline parameter values discussed above (Table 1) were run initially and the results were averaged. To use the model to help indicate relative effectiveness of some control measures, such as changing the duration of hospitalization, increasing the hospital staff, increasing the frequency of surface decontamination etc., we further ran 600 simulations of our model with different combinations of a range of parameter values (Table 2). For this work parameter values were randomized after every two simulations resulting in 280 unique combinations of parameter values out of a total of 16,384 possible combinations.

**Table 2:** Variations in model parameters.

Parameter	Variations			
Average length of stay	3 days	6 days	9 days	12 days
Average disinfection/decontamination time for HCW and TP	30 min	60 min	120 min	240min
Number of HCW	15	30	45	60
Probability of colonization of patients given contact with contaminated HCW/TP	0.02	0.04	0.06	0.08
Probability that HCW/TP get disinfected/decontaminated after average contamination period	0.9	0.8	0.7	0.6
Fraction of infected patients detected and given antibiotics	0.9	0.8	0.7	0.6
Starting day of corrected antibiotic therapy	day 1	day 2	day 3	day 4

HCW- healthcare worker, TP- transmission point.

### Environmental Survey

To compare the model results with the actual contamination prevalence inside the hospital, we conducted environmental sampling at four locations (exam rooms, the diagnostics room, ICU and the housing wards) in the hospital to estimate the fraction of time these areas were contaminated with *Enterococcus* spp or antibiotic resistant coliforms. Five samples were taken from each area at two hour intervals (midnight, 2am, 4am and so on) for 12 sampling sessions over three weeks. Samples were collected using standard 10 inch^2^ sponges (Nasco, Fort Atkinson, Wisconsin) soaked in 30 ml LB broth (Hardy Diagnostics, Santa Maria, California). LB broth (30 ml) was added to each sample sponge and samples were enriched by incubating overnight at 37°C. After incubation 1 ml of each enriched sample was spread on mEnterococcus agar (Neogen Corporation, Lansing, Michigan) plates and incubated for 48 hrs, at the end of which presence or absence of colonies was recorded. Positive samples on mEnterococcus agar were confirmed to be *Enterococcus* sp. using the PYR-salt tolerance tests [38]. Each enriched sample (1 ml) was also spread onto MacConkey agar (Becton, Dickinson and company, Sparks, Maryland) supplemented with ampicillin (16 µg/ml) and nalidixic acid (32 µg/ml) to select for Gram-negative bacteria that were resistant to both antibiotics (Amp-Nal) and incubated overnight. Average percentage contamination of each area over a 24 hour period and average percentage contamination for all four areas at the time of sampling was calculated.

### Statistical Analysis

Following simulations with different parameter sets the mean fraction of patients in the hospital that were colonized with a resistant strain and/or a non-resistant strain was evaluated using multivariate linear regression analysis. Pair-wise comparisons of individual parameters were used to determine the trend of increase or decrease in the mean fraction of patients in the hospital found to be colonized with a resistant and/or non-resistant strain due to an increase or decrease in the parameter value, in effect a sensitivity analysis to determine effects of parameters. Unpaired t-tests were used to compare the percent of times each transmission point remained contaminated according to the model and according to the environmental survey. Results from the environmental survey were also subjected to two-way ANOVA to identify associations between the level of contamination and the transmission points sampled. All statistical analyses were done in SAS analytics software (SAS Inst., Cary, North Carolina)

## Results

### Results from 500 simulations using baseline parameters

**Figure 2:**
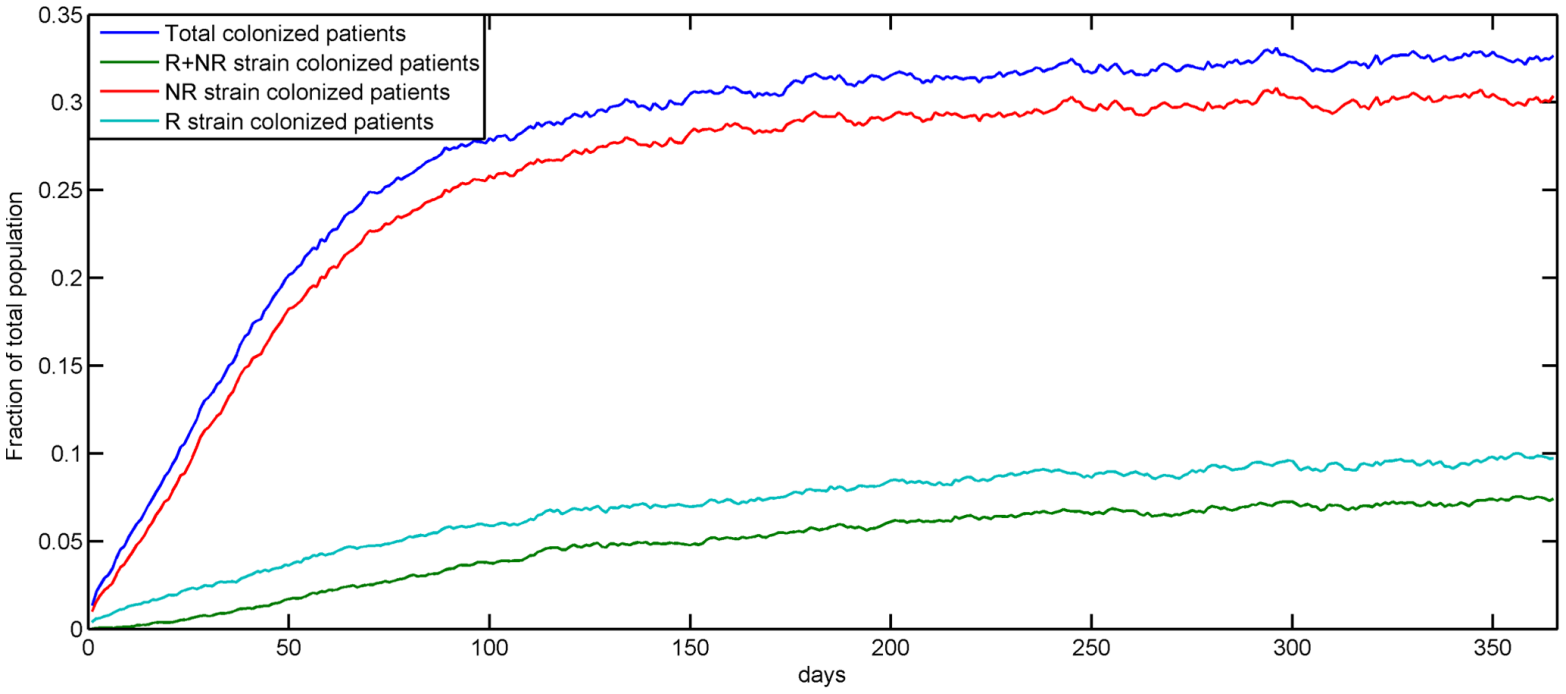
Distribution of strain types and colonization or infection status in the patient population. The fraction of patients in the hospital colonized, colonized with a resistant strain (R), colonized with a non-resistant strain (NR) and colonized with both resistant and non-resistant strains (R+NR), at the end of each day, averaged over 500 simulations.

We start the simulation for a year with a clean and empty hospital and as inpatients accumulate, the mean population of patients inside the hospital at any time reaches a stable level. At the baseline values for all parameters, the fraction of the hospital patient population colonized with any strain stabilizes at approximately 32%. Approximately 30% of the patients are colonized with the non-resistant strain, 7% are colonized with the resistant strain, and 5% are colonized with both strains (Fig. 2).

**Figure 3:**
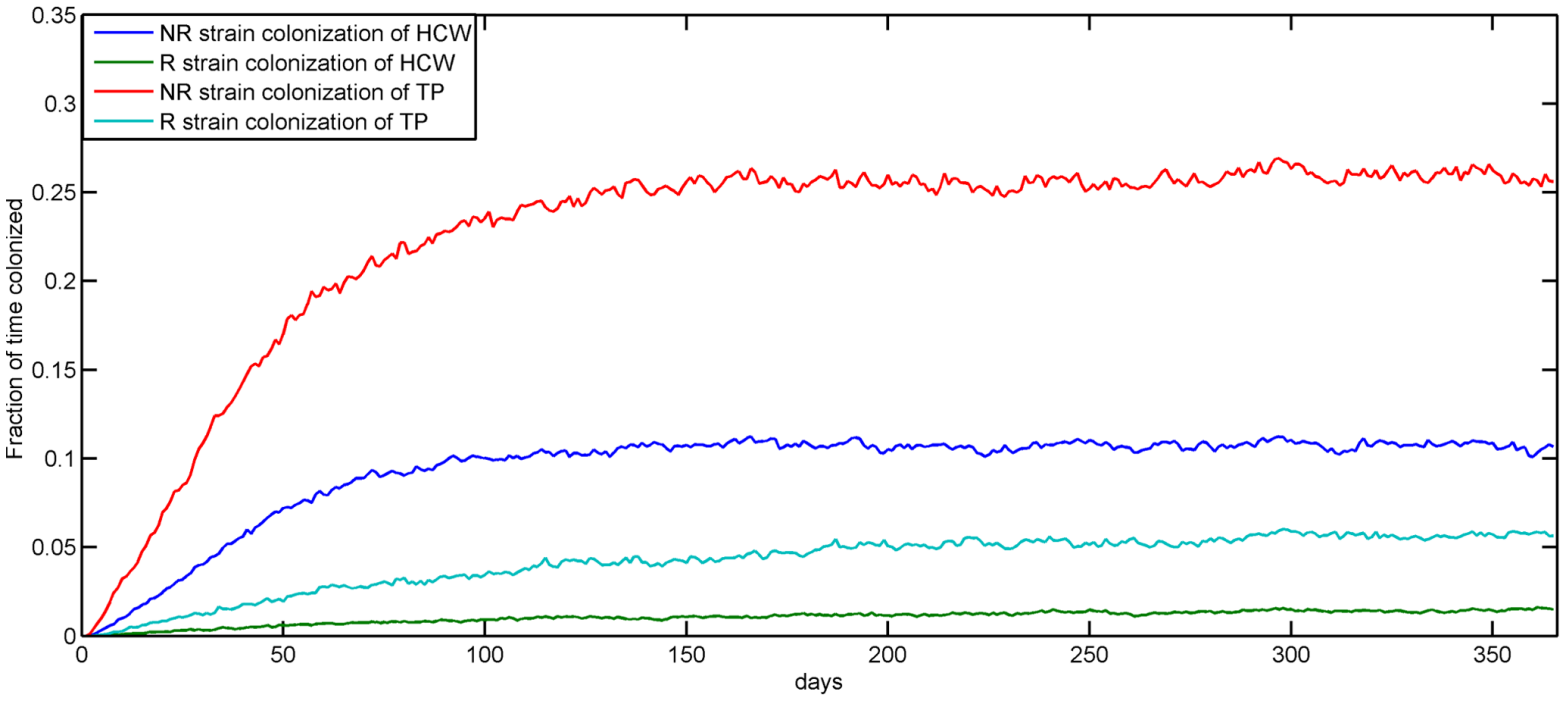
Contamination durations of healthcare workers and transmission points. The fraction of time healthcare workers (HCW) and transmission points (TP) remain contaminated with non-resistant (N) and resistant (R) strains each day averaged over 500 simulations.

Though the average time interval between decontamination of transmission points and disinfection of healthcare workers is assumed to be the same (60 min), the transmission points are contaminated with both resistant and non-resistant strains for longer durations overall (*P*<0.0001, unpaired t-test) throughout the year (Fig. 3).

**Figure 4:**
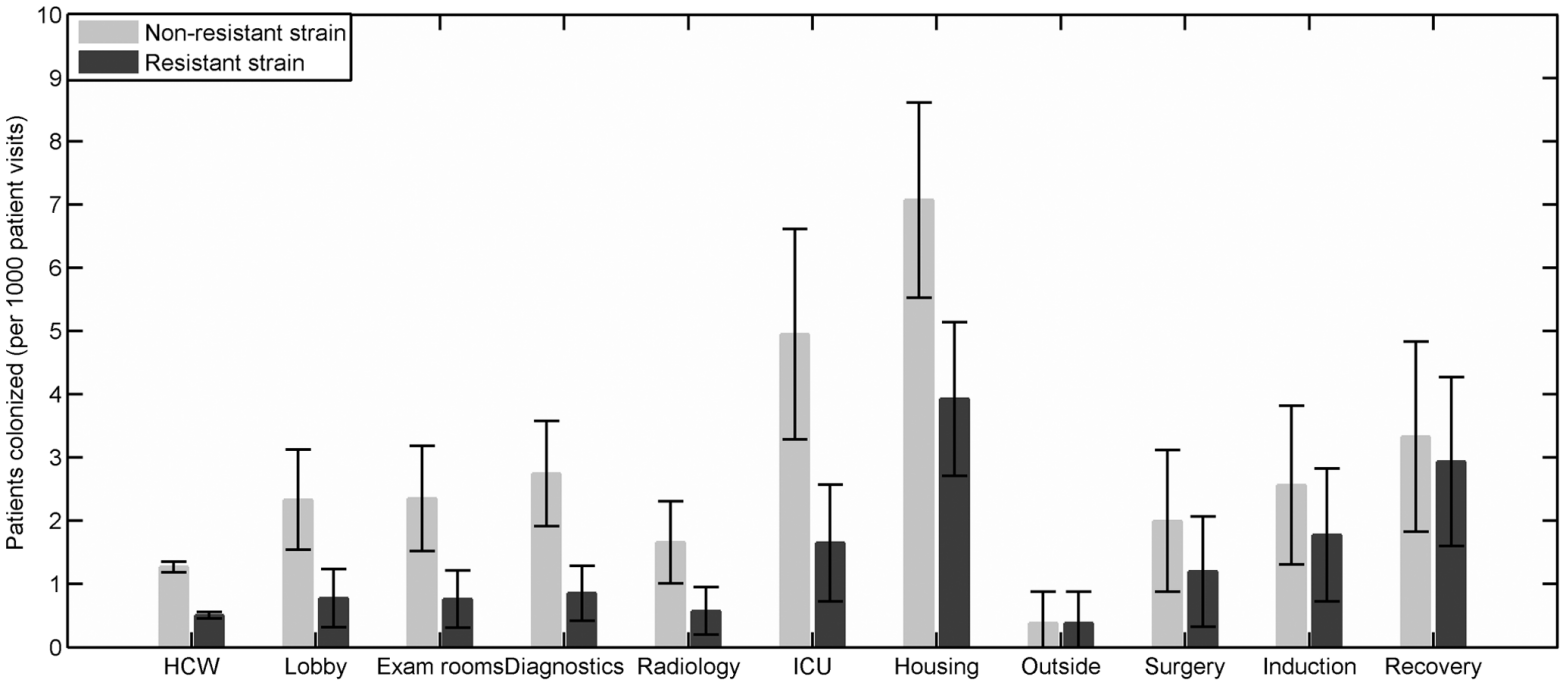
Proportion of visiting patients colonized. The average proportion of patients in contact with the healthcare workers (HCW) and the various transmission points that become colonized with non-resistant (N) and resistant (R) strains over the length of a year averaged over 500 simulations. Bars represent standard deviation across the yearly averages of 500 simulations.

**Figure 5:**
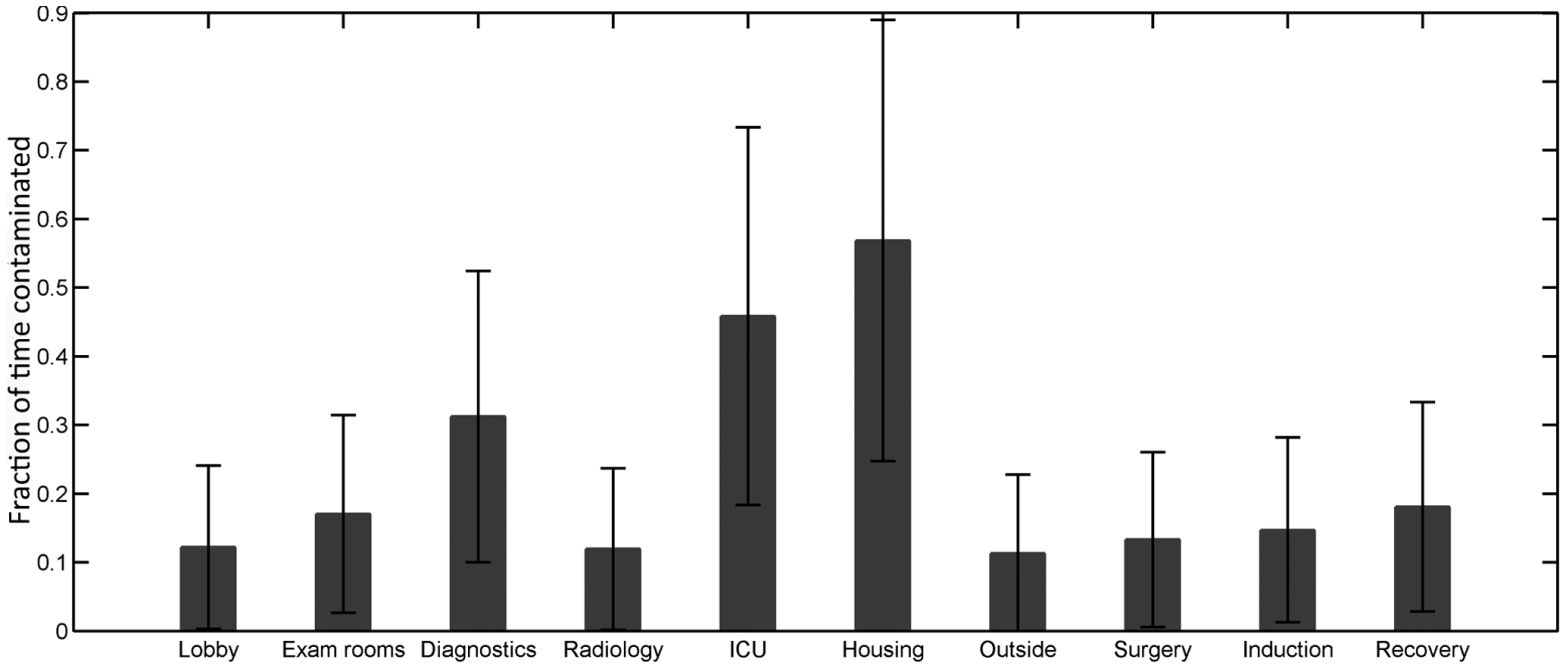
The yearly average of fraction of time that the transmission points remain contaminated. The yearly average of fraction of time each transmission point remains contaminated, averaged further over 500 simulations. Bars represent standard deviation across the yearly averages of 500 simulations.

Patient visits in the housing wards and the ICU area lead to colonization of the largest proportions of patients by the non-resistant strain. This proportion is significantly higher for housing than for all other transmission points except the ICU. The proportion of patients colonized by the resistant strain is highest for patients visiting the housing area or the recovery room. This proportion is significantly higher for housing, the ICU and diagnostics than for the surgery, exam rooms, lobby, radiology, and outside dog-walking areas (Fig. 4). The average fraction of time these places remain contaminated is also significantly higher amongst all the transmission points considered in the model, with diagnostics remaining contaminated for 31.2% (SD: 21.2%) of the time, ICU for 45.8% (SD: 27.4%) of the time and the housing wards for 56.9% (SD: 33.1%) of the time (Fig. 5).

**Figure 6:**
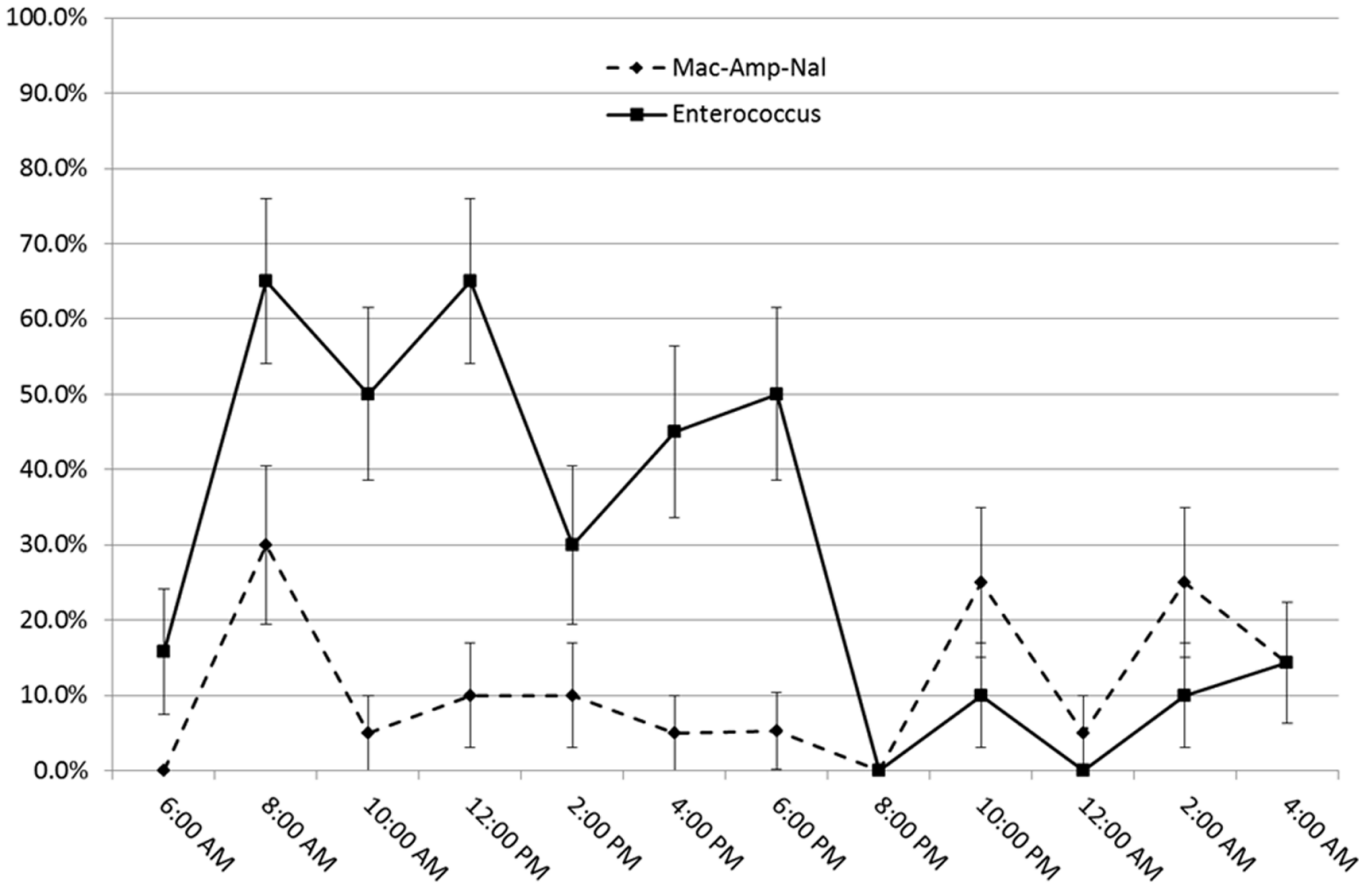
Percentage contamination with *Enterococci* or ampicillin-nalidixic acid-resistant coliforms of four transmission points by time of day. The average combined contamination prevalence of the four places sampled during the validation survey: the exam rooms, the diagnostics, ICU and the housing wards, at different times the sampling was done. Each data point is the average percentage contamination in 20 samples (5 samples per location) for each time. Bars represent the standard error over these 20 samples.

To determine the accuracy of our model predictions for the relative fractions of time that transmission points were contaminated, an environmental survey was conducted. Each of the four sampled locations (diagnostics room, exam rooms, ICU and housing wards) were frequently contaminated with *Enterococcus* spp: 27 to 35% of samples were positive. With respect to Amp-Nal coliforms, the diagnostics room, exam rooms, the ICU and the housing wards samples had 11.6, 6.7, 10.0 and 16.9 percent positive samples, respectively. The overall sample prevalence of *Enterococcus* spp. and Amp-Nal coliforms was not significantly different between locations (*Enterococcus* spp. contamination: *P* = 0.71 and coliform contamination: *P* = 0.44), although there were more positive samples for both types of bacteria in the housing wards, the ICU and the diagnostics room than in the exam rooms (Table 3). The prevalence of *Enterococcus-*positive samples was significantly higher in the daytime hours for each location but this day-night difference was not significant for Amp-Nal coliforms (Table 3 and Fig. 6).

**Table 3:** Environmental survey results.

			*Enterococcus* spp.				Amp-Nal Coliforms		
Location^a^	Day^b^	Night^b^		*P* c	Tota^d^	Day^b^	Night^b^	*P* c	Total^e^
Diagnostics Room	60.0	10.0		<0.0001	35.0	13.3	10.0	>0.99	11.6
Exam rooms	43.3	10.0		0.007	26.7	3.3	10.0	0.61	6.7
ICU	50.0	10.0		0.008	30.0	16.7	3.3	0.19	10.0
Housing wards	50.0	3.3		<0.0001	26.7	10.3	23.3	0.30	16.9

a Each location was visited a total of 12 times with 5 samples collected at each visit for a total of 30 samples during the day and 30 during the night.

b Day includes the hours between 8 AM and 8 PM. Night includes the hours between 8:00 PM and 8:00 AM. Samples were collected at 2-hour intervals.

c Fisher exact *P*-value for the difference in proportion between day and night.

d *P*-value for difference between four mean proportions  = 0.71.

e *P*-value for difference between four mean proportions  = 0.44.

### Effects of Varying Parameters of the Model

Regression analysis of the mean fraction of patients colonized with a resistant strain produced a statistically significant fit (*P*<0.001) with an R^2^ = 0.957. The Type III sums of squares indicated that the average length of stay parameter explained the bulk of the variance in the model with probability of colonization given contact, average time to disinfection of healthcare workers and decontamination of transmission points, and number of healthcare workers in the hospital also being significant in descending order of importance. The starting day of effective antibiotic therapy, efficiency of disinfection of healthcare workers and decontamination of transmission points, and infection detection rate were not significant (Table 4).

**Table 4:** Type III statistical test results for analysing the significance of various parameters on the mean fraction of the patient population carrying the resistant strain of the potential pathogen.

Source	Type III SS	Mean Square	F Value	Pr>F
Length of stay^a^	8.0319	2.6773	1283.02	<.0001
Detection rate^b^	0.01614	0.00538	2.58	0.054
Decontamination efficiency^c^	0.01143	0.00381	1.83	0.1425
Decontamination time^d^	1.23757	0.41252	197.69	<.0001
Colonization probability^e^	2.0568	0.6856	328.55	<.0001
Starting day of AB therapy^f^	0.00698	0.00233	1.11	0.3434
Number of HCW^g^	0.21583	0.07194	34.48	<.0001

a Average length of stay of hospitalized patients.

b Rate at which infections are detected.

c Efficiency of disinfection/decontamination of healthcare worker and transmission points.

d Average time before disinfection/decontamination of contaminated healthcare worker and transmission point.

e Probability of colonization of patient given contact with contaminated healthcare workers and transmission points.

f Number of days after the initial antibiotic therapy that the effective antibiotic therapy starts.

g Number of healthcare workers inside the hospital at any given time.

Regression analysis of the mean fraction of patients colonized with a non-resistant strain also produced a statistically significant fit (*P*<0.001) with an R^2^ = 0.965. The average length of stay parameter again explained most of the variance in the model with probability of colonization given contact, average time of disinfection of healthcare worker and decontamination of transmission point, and number of healthcare workers in the hospital also significant in descending order of importance. The rate of infection detection was also a significant contributor to the mean fraction of the patient population that was colonized with a non-resistant stain (Table 5).

**Table 5:** Type III statistical test results for analysing the significance of various parameters on the mean fraction of the patient population carrying the non-resistant strain of the potential pathogen.

Source	Type III SS	Mean Square	F Value	Pr>F
Length of stay^a^	5.81725	1.93908	1483.7	<.0001
Detection rate^b^	0.23787	0.07929	60.67	<.0001
Decontamination efficiency^c^	0.00156	0.00052	0.4	0.7554
Decontamination time^d^	1.1509	0.38363	293.54	<.0001
Colonization probability^e^	1.58922	0.52974	405.33	<.0001
Starting day of AB therapy^f^	0.00474	0.00158	1.21	0.3072
Number of HCW^g^	0.14332	0.04777	36.55	<.0001

a Average length of stay of hospitalized patients.

b Rate at which infections are detected.

c Efficiency of disinfection/decontamination of healthcare worker and transmission points.

d Average time before disinfection/decontamination of contaminated healthcare worker and transmission point.

e Probability of colonization of patient given contact with contaminated healthcare workers and transmission points.

f Number of days after the initial antibiotic therapy that the effective antibiotic therapy starts.

g Number of healthcare workers inside the hospital at any given time.

Least square means analysis was used to make pairwise comparisons between the average fractions of the population colonized with the resistant strain and/or non-resistant strain for a range of parameter values. There was a significant increase in the mean fraction of the patient population colonized with increasing length of stay (*P*<0.0001 in all cases) and a consistent decrease in the fraction of population colonized with the non-resistant strain with increases in the detection rate (*P*<0.0001 in all cases). There was a significant increase in the mean fraction of the patient population colonized with increasing duration of TP or HCW contamination (*P*<0.0001 in all cases). Analysis of maximum likelihood parameter estimates for the interaction between increasing the mean time of disinfection/decontamination with a change in transmission point showed that the lobby, exam rooms and diagnostic area have significantly greater increases in proportions of visiting patients that are colonized with resistant and/or non-resistant strains as compared to the housing wards and ICU. Increasing the probability of colonization of a patient given contact (analogous to a hand hygiene lapse) caused a significant increase in the mean fraction of the patient population colonized with (*P*<0.0001 in all cases). There was a significant increase in the mean fraction of patient population colonized given an increasing number of healthcare workers from 15 to 30, but increasing beyond this level did not alter the outcome (*P*>0.05) (Table S1, Table S2).

## Discussion

Model simulations done at baseline parameter values indicate that specific transmission points in the hospital such as the ICU, the housing wards and the recovery room, have more influence on transmission of colonization, including transmission of resistant strains, than other locations in the hospital. In the model, the housing and the ICU areas are associated with more transmissions due to the relatively long stays of hospitalized patients in these places [39], particularly at night. The proportions of time that housing wards were contaminated with Amp-Nal resistant coliforms during the environmental survey support this speculation (23.3% at night as compared to 10.3% during the day). However, for *Enterococcus* spp. the reverse was true (Fig. 6). Among the transmission points exclusive for surgery patients, the recovery room had as much impact on non-resistant strain colonization of patients as the diagnostics room and a greater impact in the case of resistant strain colonization. This may be due to the fact that surgery-related transmission points are visited by patients that stay for longer durations in the hospital as compared to most of the patients visiting the diagnostics area. In the model, this allows for increased bacterial loads among surgery patients, leading to a larger probability of contamination of healthcare workers and the transmission points they visit and eventually increased chances of uncolonized visiting patients getting colonized. In general, the transmission points are contaminated for longer durations as compared to healthcare workers (Fig. 3) and cause colonization of more patients visiting them (Fig. 4). This is mainly because they are assumed to have multiple patient and healthcare worker visits in the model. But in terms of the absolute number of patients colonized, the effect of transmission points and healthcare workers are comparable due to relatively more frequent patient contacts by healthcare workers as compared to transmission points (Table 1).

Results from the simulations done with randomized parameter values and subsequent regression analyses suggest that the model is consistent with previous reports that the incidence of both resistant and non-resistant strain colonization increases with increasing length of stay in the hospital [14], [15], [33]. As long as the number of patients already hospitalized is below the maximum capacity for the hospital, longer patient stays contribute to a higher number of hospitalized patients, leading to more interactions between patients, healthcare workers and transmission points. Each individual patient also has a greater possibility of getting colonized during a longer stay in the hospital. Veterinary personnel and veterinary hospital environments are reportedly major risk factors in acquisition of antibiotic resistant pathogens by hospitalized dogs [15], [40], [41]. Consequently, reducing the probability of colonization of a patient given contact with a contaminated healthcare worker or transmission point reduces the percent of patients carrying resistant and/or non-resistant strains. The probability of colonization given contact can be reduced by increasing healthcare worker hand hygiene compliance and by improved cleaning and disinfection of hospital outerwear such as scrubs and white coats [42]. A decrease in the frequency of disinfection/decontamination of the healthcare workers and the transmission points resulted in a general increase in the number and incidence of nosocomial colonization in our model. This effect was most pronounced in the lobby, exam room and diagnostics areas suggesting that those places require more frequent cleaning, possibly because of higher traffic load during the daytime, as compared to the ICU and housing. This idea is supported by the findings of our environmental survey in which prevalence of contamination with *Enterococci* was higher during the day than during the night. In contrast, Amp-Nal coliform sample prevalence was not different between the day and nighttime hours. The reason for this difference is unknown. Coliforms are less persistent on surfaces in general [43], and therefore the sampling scheme here may not have fully captured their spatial-temporal distribution.

Increasing the number of healthcare workers might lead to safer interactions between patients and healthcare workers because caregivers would have fewer opportunities to cross-contaminate patients, but in our model it also increases the number of interactions between transmission points and healthcare workers leading to more contamination of transmission points and hence no significant change in the mean proportion of patients colonized. If the number of healthcare workers is very low as compared to the average patient population at any time, there is a significant decrease in the percent of patients that are colonized. This presumably occurs due to overall fewer interactions between patients and healthcare workers during their stay. Decreasing the number of healthcare workers is not pragmatic as it would increase the workload on individual healthcare workers and may lead to deterioration of care.

It is well documented that antimicrobial use is associated with antibiotic resistance [6], [14], [33], [44], but our model indicates that giving an early effective antibiotic therapy has no significant impact on either reducing or increasing the incidence of antibiotic resistance. This might be a result of the relatively short average length of stay of the patients and shorter time available for antibiotic therapy completion.

The bacterial species considered in this model are enteric bacterial pathogens like *Enterococcus* spp., *E. coli* and *K. pneumoniae*. All three of these fecal organisms can spread to patients due to surface to body contact, followed by oral ingestion. *Enterococcus* spp. generally survive longer in the environment than gram-negative bacteria do which may explain their near ubiquitous presence in our environmental survey. But their presence provides an indication of fecal contamination and inadequate cleaning and disinfection [39]. Gram-negative bacteria provide evidence for more recent fecal contamination [43]. The proportion of time of contamination with Amp-Nal coliforms during the environmental survey better reflected the predictions of the model, although no significant differences were found between the different places sampled. The model does fail to explain the higher level of *Enterococcus* spp. contamination during daytime than during nighttime hours. A possible explanation for the difference between Enterococci and Amp-Nal resistant coliforms is that the coliforms were specifically selected for resistance to antibiotics which may have co-selected for resistance to disinfectants. Thus after evening disinfection and during low traffic hours the Enterococci were less likely to be reintroduced by patient traffic. The model also indicates that places with shorter visits and higher patient traffic of patients (e.g., the exam room and the lobby) require more frequent disinfection/decontamination.

This model provides a significant contribution to the field of hospital modeling because it accounts for individual patient movements through the hospital rather than assuming a strictly compartmental structure of patient movements. The primary purpose of this effort was to generate a conceptual framework for predicting changes in antimicrobial resistant bacterial transmission in response to changes in the chosen parameters. The determination of baseline parameter values was limited because of a lack of empirical data; for example the true probability of initial colonization and infection given a previous colonization is not known. Nonetheless the relative effect of changing a parameter, for example changing the average length of hospital stay, is unlikely to be biased by the choice of a baseline. While intensive sampling for more empirically based parameterization would strengthen the model, such sampling was beyond the scope of the current effort. This will be included in future work to refine and expand on this model. Regardless of specific parameter values, the sensitivity analysis provides information about which variables will have the most impact and therefore where interventions should be targeted.

In summary, this model suggests that reducing the average length of stay of patients and more frequent disinfection of healthcare workers and decontamination of transmission points are the most important control measures to minimize nosocomial transmission and frequency of colonization or infection with resistant strains inside the hospital. Extensions of this model, such as considering multiple patient and pathogen species, variable healthcare worker population and using empirical data as a basis for the transmission probability estimates used here may give further insight into the risk factors associated with the spread of antibiotic resistance in veterinary hospitals with availability of extensive hospital data.

## Supporting Information

Table S1 Results of least square means comparisons between the average fractions of the population colonized with the resistant strain for different parameter values.
(PDF)

Table S2 Results of least square means comparisons between the average fractions of the population colonized with the non-resistant strain for different parameter values.
(PDF)
