# Supplementary material for: An Individual-Based Model of Transmission of Resistant Bacteria in a Veterinary Teaching Hospital
Source: PLoS One. 2014 Jun 3;9(6):e98589. doi: 10.1371/journal.pone.0098589 (PMC4043964; doi:10.1371/journal.pone.0098589)
Supplement: Table S1 — Results of least square means comparisons between the average fractions of the population colonized with the resistant strain for different parameter values. (PDF) [file pone.0098589.s001.pdf]

Differences of Least Squares Means using fraction of patient population colonized with resistant strain

Effect	als	dr	deconefficiency	decon	pcptc	et	nbh	_als	_dr	_deconefficiency	_decon	_pcptc	_et	_nbh	Estimate	Standard Error	DF	t Value	Pr > |t|
als	3							6							-0.2207	0.0087	278	-25.37	<.0001
als	3							9							-0.3694	0.00793	278	-46.57	<.0001
als	3							12							-0.4685	0.00798	278	-58.68	<.0001
als	6							9							-0.1487	0.00802	278	-18.55	<.0001
als	6							12							-0.2478	0.00772	278	-32.12	<.0001
als	9							12							-0.09907	0.00703	278	-14.09	<.0001
dr		0.6							0.7						-0.01695	0.00792	278	-2.14	0.0331
dr		0.6							0.8						-0.01737	0.00798	278	-2.18	0.0304
dr		0.6							0.9						-0.0196	0.0078	278	-2.51	0.0125
dr		0.7							0.8						-0.00042	0.00783	278	-0.05	0.9573
dr		0.7							0.9						-0.00264	0.00749	278	-0.35	0.7244
dr		0.8							0.9						-0.00222	0.00772	278	-0.29	0.7735
deconefficiency			0.6							0.7					-0.0118	0.0078	278	-1.51	0.1315
deconefficiency			0.6							0.8					0.00554	0.00779	278	0.71	0.4774
deconefficiency			0.6							0.9					0.00131	0.00761	278	0.17	0.8629
deconefficiency			0.7							0.8					0.01734	0.00777	278	2.23	0.0264
deconefficiency			0.7							0.9					0.01311	0.00769	278	1.7	0.0893
deconefficiency			0.8							0.9					-0.00423	0.00767	278	-0.55	0.5819
decon				30							60				-0.07355	0.00767	278	-9.59	<.0001
decon				30							120				-0.1321	0.00775	278	-17.05	<.0001
decon				30							240				-0.177	0.00774	278	-22.86	<.0001
decon				60							120				-0.05851	0.00783	278	-7.48	<.0001
decon				60							240				-0.1035	0.00771	278	-13.42	<.0001
decon				120							240				-0.045	0.00793	278	-5.68	<.0001
pcptc					0.02							0.04			-0.1136	0.00742	278	-15.31	<.0001
pcptc					0.02							0.06			-0.1733	0.00744	278	-23.3	<.0001
pcptc					0.02							0.08			-0.2181	0.00755	278	-28.9	<.0001
pcptc					0.04							0.06			-0.05973	0.00784	278	-7.62	<.0001
pcptc					0.04							0.08			-0.1045	0.00794	278	-13.17	<.0001
pcptc					0.06							0.08			-0.04482	0.00805	278	-5.57	<.0001
et						1							2		-0.00926	0.00821	278	-1.13	0.2603
et						1							3		0.00155	0.00743	278	0.21	0.8349
et						1							4		-0.00919	0.00743	278	-1.24	0.2168
et						2							3		0.01081	0.00818	278	1.32	0.1874
et						2							4		6.7E-05	0.0083	278	0.01	0.9935
et						3							4		-0.01074	0.00756	278	-1.42	0.1564
nbh							15							30	-0.05666	0.00742	278	-7.64	<.0001
nbh							15							45	-0.05851	0.00755	278	-7.75	<.0001
nbh							15							60	-0.06984	0.00786	278	-8.89	<.0001
nbh							30							45	-0.00185	0.00754	278	-0.25	0.8062
nbh							30							60	-0.01318	0.00784	278	-1.68	0.0938
nbh							45							60	-0.01133	0.00811	278	-1.4	0.1632

Legend
Als- average length of stay of patients in days
Decon- average time of decontamination of hcw and tp in minutes
Decon efficiency- efficiency of decontamination of hcw and tp at the end of contamination period
Et- starting day of corrected antibiotic therapy
Dr- detection rate of infection
Nbh- number of hcw in hospital
Pcptc- probability of colonization of patient given contact with contaminated tp/hcw
