# Supplementary material for: An Individual-Based Model of Transmission of Resistant Bacteria in a Veterinary Teaching Hospital
Source: PLoS One. 2014 Jun 3;9(6):e98589. doi: 10.1371/journal.pone.0098589 (PMC4043964; doi:10.1371/journal.pone.0098589)
Supplement: Table S2 — Results of least square means comparisons between the average fractions of the population colonized with the non-resistant strain for different parameter values. (PDF) [file pone.0098589.s002.pdf]

Differences of Least Squares Means using fraction of patient population colonized with non-resistant strain

Effect	als	dr	deconefficiency	decon	pcptc	et	nbh	_als	_dr	_deconefficiency	_decon	_pcptc	_et	_nbh	Estimate	Standard Error	DF	t Value	Pr > |t|
als	3							6							-0.2013	0.00688	278	-29.24	<.0001
als	3							9							-0.3222	0.00628	278	-51.32	<.0001
als	3							12							-0.3994	0.00632	278	-63.21	<.0001
als	6							9							-0.1209	0.00635	278	-19.05	<.0001
als	6							12							-0.1981	0.00611	278	-32.44	<.0001
als	9							12							-0.07722	0.00557	278	-13.87	<.0001
dr		0.6							0.7						0.01228	0.00627	278	1.96	0.0511
dr		0.6							0.8						0.03924	0.00632	278	6.21	<.0001
dr		0.6							0.9						0.07505	0.00617	278	12.16	<.0001
dr		0.7							0.8						0.02697	0.0062	278	4.35	<.0001
dr		0.7							0.9						0.06277	0.00593	278	10.59	<.0001
dr		0.8							0.9						0.03581	0.00611	278	5.86	<.0001
deconefficiency			0.6							0.7					-0.00319	0.00617	278	-0.52	0.6057
deconefficiency			0.6							0.8					-0.00045	0.00616	278	-0.07	0.9422
deconefficiency			0.6							0.9					0.00341	0.00602	278	0.57	0.5717
deconefficiency			0.7							0.8					0.00274	0.00615	278	0.45	0.6558
deconefficiency			0.7							0.9					0.0066	0.00609	278	1.08	0.2792
deconefficiency			0.8							0.9					0.00386	0.00607	278	0.64	0.5258
decon				30							60				-0.06247	0.00607	278	-10.3	<.0001
decon				30							120				-0.1214	0.00613	278	-19.8	<.0001
decon				30							240				-0.1708	0.00613	278	-27.88	<.0001
decon				60							120				-0.05894	0.00619	278	-9.52	<.0001
decon				60							240				-0.1084	0.0061	278	-17.76	<.0001
decon				120							240				-0.04944	0.00627	278	-7.88	<.0001
pcptc					0.02							0.04			-0.0932	0.00587	278	-15.87	<.0001
pcptc					0.02							0.06			-0.1494	0.00589	278	-25.38	<.0001
pcptc					0.02							0.08			-0.193	0.00597	278	-32.31	<.0001
pcptc					0.04							0.06			-0.05619	0.0062	278	-9.06	<.0001
pcptc					0.04							0.08			-0.09976	0.00628	278	-15.88	<.0001
pcptc					0.06							0.08			-0.04358	0.00637	278	-6.84	<.0001
et						1							2		-0.00646	0.0065	278	-0.99	0.3207
et						1							3		3.8E-05	0.00588	278	0.01	0.9948
et						1							4		-0.00921	0.00588	278	-1.57	0.1182
et						2							3		0.0065	0.00647	278	1	0.3162
et						2							4		-0.00275	0.00657	278	-0.42	0.676
et						3							4		-0.00925	0.00598	278	-1.55	0.1233
nbh							15							30	-0.04615	0.00587	278	-7.86	<.0001
nbh							15							45	-0.04571	0.00597	278	-7.65	<.0001
nbh							15							60	-0.05808	0.00622	278	-9.34	<.0001
nbh							30							45	0.00045	0.00597	278	0.07	0.9403
nbh							30							60	-0.01193	0.0062	278	-1.92	0.0555
nbh							45							60	-0.01238	0.00642	278	-1.93	0.0547

Legend

Als- average length of stay of patients in days

Decon- average time of decontamination of hcw and tp in minutes

Decon efficiency- efficiency of decontamination of hcw and tp at the end of contamination period

Et- starting day of corrected antibiotic therapy

Dr- detection rate of infection

Nbh- number of hcw in hospital

Pcptc- probability of colonization of patient given contact with contaminated tp/hcw
